# Soft fibrin matrix instructs macrophage M2 polarization via FABP4-mediated enhancement of fatty acid β-oxidation to alleviate MASH

**DOI:** 10.1007/s00018-026-06266-2

**Published:** 2026-06-04

**Authors:** Ranran Zhang, Jialong Liu, Wenqing Shan, Haizhou Wang, Liping Gao, Yizhang Li, Lilan Fan, Chaoran Yang, Guoqingyuan Li, Jing Liu

**Affiliations:** 1 Department of Gastroenterology, Zhongnan Hospital of Wuhan University, Wuhan, 430071 China; 2 Department of Gastroenterology, Shandong Medical and Pharmaceutical University Hospital, Binzhou, 256600 China; 3 Hubei Provincial Clinical Research Center for Intestinal and Colorectal Diseases, Hubei Key Laboratory of Intestinal and Colorectal Diseases, Wuhan, China; 4 Department of Gastroenterology, Zhongnan Hospital of Wuhan University, No. 169 Donghu Road, Wuchang District, Wuhan, 430071 China

**Keywords:** Metabolic dysfunction-associated steatohepatitis, FABP4, Macrophage immunometabolism, Fibrin matrix, PPARα

## Abstract

**Background:**

Macrophage-driven inflammation is a key pathogenic factor in metabolic dysfunction-associated steatohepatitis (MASH). While fibrin deposition is observed in MASH livers, its role in regulating macrophage polarization and disease progression remains unclear.

**Methods:**

We investigated fibrin deposition in liver tissues from MASH patients and high-fat diet (HFD)-induced MASH mice. Macrophages were cultured in soft or stiff 3D fibrin matrices to assess polarization phenotypes. In vivo, macrophages pre-cultured in soft or stiff fibrin were adoptively transferred into HFD-fed mice. Mechanistic studies involved siRNA-mediated knockdown, overexpression, chromatin immunoprecipitation, co-immunoprecipitation, and metabolomic analyses. Macrophage-specific FABP4 knockdown was achieved using an AAV8 vector expressing F4/80-driven FABP4 shRNA and eGFP.

**Results:**

Fibrin deposition was significantly elevated in MASH livers and correlated with pro-inflammatory macrophage infiltration. Macrophages cultured in soft fibrin matrices exhibited enhanced anti-inflammatory (M2) polarization, characterized by increased Arg1 and CD206 and decreased iNOS and TNFα expression. Adoptive transfer of soft fibrin-cultured macrophages alleviated hepatic steatosis, insulin resistance, and oxidative stress in MASH mice. Mechanistically, soft fibrin downregulated FABP4 expression by inhibiting ITGB2/STAT1 signaling. FABP4 competitively bound to PPARα, thereby sterically hindering the access of USP53, which functions as a deubiquitinase, to PPARα. This displacement prevented USP53-mediated deubiquitination, resulting in increased PPARα ubiquitination and proteasomal degradation, ultimately leading to suppression of fatty acid β-oxidation. Macrophage-specific FABP4 knockdown mirrored the therapeutic effects of soft fibrin, ameliorating MASH pathology.

**Conclusions:**

Our findings reveal that soft fibrin matrices promote anti-inflammatory macrophage polarization and mitigate MASH by enhancing fatty acid β-oxidation via downregulation of FABP4 and stabilization of PPARα. Targeting the Fibrin-FABP4-PPARα axis may offer a novel therapeutic strategy for MASH.

**Supplementary Information:**

The online version contains supplementary material available at 10.1007/s00018-026-06266-2.

## Introduction

Metabolic dysfunction-associated steatotic liver disease (MASLD) affects 25% of the global population and currently lacks an approved treatment [1]. Metabolic dysfunction-associated steatohepatitis (MASH), an advanced form of MASLD, is one of the leading causes of organ transplantation and liver cancer [2, 3]. Its pathophysiology involves lipotoxicity, hepatic immune disturbances, and hepatic insulin resistance [4]. Given the complexity of MASH pathophysiology, single-molecule targeted therapeutics, the foundation of modern drug development, have been tested in MASH clinical trials with limited success [5, 6]. In recent years, the application of cell therapy for liver diseases has been frequently reported and may present a novel therapeutic approach for these conditions. For example, Mesenchymal stem cell therapy can improve survival in patients with HBV-related decompen sated liver cirrhosis [7]. In addition, Autologous macrophage therapy in patients with compensated cirrhosis reduce inflammation, resolve fibrosis and stimulate liver regeneration [8]. Macrophage activation in hepatic sinusoids plays a crucial role in the pathogenesis of MASLD due to its pleiotropic characteristics [9, 10]. Circulating monocytes may be the most important macrophages mediating liver inflammation [11]. Macrophages are highly adaptable and can polarize into different phenotypes under various cellular microenvironmental stimuli. They polarize into the classic M1 or alternatively activated M2 phenotypes. M1 macrophages highly express proinflammatory genes, including TNFα, IL-1β, and inducible nitric oxide synthase (iNOS), while M2 macrophages exhibit anti-inflammatory properties through the expression of arginase 1 (Arg-1) and CD206. An imbalance between M1 and M2 macrophages is closely associated with MASLD progression. Reversing macrophage polarization from M1 to M2 through annexin A5 targeting has shown a protective effect against MASH [12]. Preventing lipid accumulation in macrophages could inhibit the transition to a proinflammatory phenotype and the release of cytokines such as TNFα [13]. These findings suggest that identifying key regulatory factors involved in macrophage polarization and activation could offer a promising approach for MASH treatment.

Fibrin is a protein involved in blood clotting and is a key component of the provisional extracellular matrix formed following tissue injury [14]. As an extracellular matrix, fibrin could binds to integrin molecules on the cell surface via the arginine-glycine-aspartate sequence, mediating cell adhesion, differentiation, migration, inflammation, and other signaling processes [15, 16]. Fibrin deposition in the hepatic sinusoid has been shown to increase during the development of MASLD and obesity [17, 18]. Macrophages live in a highly dynamic mechanical microenvironment. How the extracellular-matrixgenerated mechanotransduction regulates macrophage activation remains an enigma.

Fatty acid-binding proteins (FABPs), such as FABP1, FABP2, FABP3, and FABP4, have been reported to be associated with metabolic and inflammatory diseases through their regulation of lipid trafficking and cellular responses [19]. FABP4 is highly expressed in adipocytes and macrophages. Moreover, recent studies have indicated that FABP4 expression is elevated in MASLD [20, 21]. In FABP4 knockout macrophages, the production of proinflammatory cytokines and enzymes, such as IL-1β, TNFα, and iNOS, is reduced [22]. Overexpression of FABP4 in macrophages can exacerbate atherosclerosis [23]. However, the relationship between FABP4 and macrophages in MASH requires further investigation.

Previously, a novel three-dimensional (3D) culture system using fibrin hydrogel was established, demonstrating that fibrin stiffness plays a crucial role in tumorigenic cell differentiation. By varying the concentrations of fibrinogen, the stiffness of fibrin gels can be easily adjusted [24, 25]. In this study, an in-house 3D fibrin culture system was utilized to investigate the effects of fibrin stiffness in macrophage activation and to explore the role of fibrin in MASH and its underlying mechanisms. We found that macrophages seeded in soft fibrin alleviated hepatic steatosis and insulin resistance in high-fat diet (HFD)-fed MASH mice. This effect of soft fibrin may be mediated by the regulation of fatty acid metabolic reprogramming in macrophages. The study reveals novel regulatory mechanisms and identifies potential therapeutic targets for MASH treatment.

## Materials and methods

### Liver samples

Liver samples were obtained from patients who underwent liver biopsy, surgery, or transplantation at the Department of Hepatobiliary Surgery, Zhongnan Hospital of Wuhan University. To evaluate the MASLD activity score, at least two pathologists independently performed H&E staining of liver sections in a blinded manner according to the MASLD activity score. Patients were excluded from this study if they had toxin- or drug-induced liver injury, excessive alcohol consumption, hepatitis virus infection, autoimmune liver diseases, genetic liver disorders (including hemochromatosis and Wilson’s disease), or active malignancy. Baseline demographic and clinical information for all enrolled subjects, including age, sex, body mass index (BMI), diabetes status, fibrosis stage, and NAFLD activity score (NAS), is provided in Supplementary Table 1. All procedures involving human sample collection were approved by the Ethics Committee of Zhongnan Hospital of Wuhan University and conducted in accordance with the principles of the Declaration of Helsinki.

## Preparation of bone marrow-derived macrophages and cell culture

C57BL/6 mice (6–8 weeks old) were sacrificed, and their femurs were dissected. A 21-gauge needle was used to flush the femurs with DMEM. After centrifugation at 400×g for 5 min at 4℃, cells were resuspended in DMEM (GIBCO, NY) supplemented with 10% fetal bovine serum, 100 U/mL penicillin-streptomycin, and 20 ng/mL M-CSF (PeproTech, USA). Mouse RAW264.7 macrophages and HEK-293 cell were cultured in DMEM medium supplemented with 10% fetal bovine serum. The mouse hepatic cell line AML12 was cultured in AML12 cell culture medium (Procell Life Science & Technology, China). Cells were maintained in a cell incubator at 37 °C under a water-saturated atmosphere with 5% CO₂.

## Cell culture in 3D fibrin gels

For 3D cell culture, fibrin gels were prepared as previously described [24]. Salmon fibrinogen and thrombin were purchased from Searun Holdings (San Diego, CA, USA). Fibrinogen and the cell suspension were mixed at a 1:1 (v/v) ratio to obtain final fibrinogen concentrations of 1 mg/mL or 4 mg/mL. Next, 250 µL of the fibrinogen-cell mixture and 5 µL of thrombin (100 U/mL) were mixed thoroughly and used to coat 24-well plates. The plates were incubated for 30 min at 37 °C in a CO₂ incubator, after which DMEM supplemented with 10% FBS was added. After 48 h, macrophages were harvested using Dispase II (Roche, Switzerland).

## Gel stiffness calculation

Fibrin gels with concentrations of 1 mg/mL and 4 mg/mL corresponded to shear moduli of 30 Pa and 140 Pa, respectively, as measured using an RFS III fluid spectrometer 50. The relationship between elastic stiffness (E) and shear modulus (G) is given by the formula G = E/2 (1 + µ), where µ is the Poisson’s ratio. The Poisson’s ratio of fibrin was 0.5, meaning that 1 mg/mL and 4 mg/mL fibrin gels. In this study, these were referred to as soft fibrin and stiff fibrin.

## Animals and reagents

All animal studies were conducted in accordance with the guidelines of the Animal Ethics Committee of Wuhan University (ZN2023227). Male C57BL/6J mice (6–8 weeks old) were purchased from Vital River Laboratory Animal Technology Co., Ltd. (Beijing, China). The mice were housed under a 12 h light/dark cycle at a temperature of 23.5 ± 2.5℃ and a humidity level of 52.5 ± 12.5%. To establish the MASH model, mice were fed a high-fat diet (HFD; protein, 14%; fat, 42%; carbohydrates, 44%; cholesterol, 0.2%; TP26304; Trophic Animal Feed High-Tech Co.,Ltd, Nantong, China), with fat mainly derived from anhydrous milk fat, for a total of 16 weeks. Before HFD feeding, mice were randomly assigned to four groups: NCD, HFD, HFD +stiff fibrin, and HFD+soft fibrin.

BMDMs or RAW264.7 macrophages were cultured in fibrin gels of different stiffness for 48 h. To harvest the cells, the culture medium was removed, the gels were gently minced using pipette tips, and 500 µL/well Dispase II (2 U/mL) was added for digestion at 37 °C for 10 min until the fibrin gels were completely dissolved. An equal volume of culture medium was then added to stop the digestion. The cell suspension was filtered through a cell strainer and centrifuged at 400×g for 5 min to collect the cell pellet. The cells were washed twice with PBS, counted, and assessed for viability and purity before tail vein injection. Macrophage treatment was initiated after 12 weeks of HFD feeding. Before intervention, one randomly selected HFD-fed mouse was subjected to histological examination to confirm establishment of the MASH phenotype based on NAS-related features, including steatosis, lobular inflammation, and hepatocellular ballooning. A total of 1 × 10^6 macrophages in 100 µL PBS were injected into each mouse via the tail vein once weekly for 4 weeks, while HFD feeding was continued until week 16. Mice were euthanized 3 days after the final injection for further analysis.

To generate macrophage-specific-FABP4 knockdown mice, AAV8 viral vectors carrying the F4/80 promoter, FABP4 shRNA, and the eGFP reporter gene were used. Briefly, the mice received an intravenous injection of AAV8-F4/80-shFABP4 for macrophage-specific FABP4 knockdown or AAV8-F4/80-shCon as the negative control (1.0 × 10^12 active viral particles were diluted in 200 µl saline per mouse; PackGene, guangzhou, China). Successful transfection and knockdown of macrophages FABP4 were confirmed 30 days post-infection by immunofluorescence, and western blotting using gradient-centrifugation-isolated macrophages, after which HFD feeding was initiated.

### Collection of liver tissues and blood samples

Mice were euthanized by intraperitoneal injection of pentobarbital, and blood samples were collected. The livers were weighed, and liver tissues were used for subsequent experiments. Serum was separated by centrifuging blood samples at 3000×g for 15 min at 4 °C. Liver function-related parameters, including Alanine Aminotransferase (ALT), Aspartate Aminotransferase, malondialdehyde (MDA), Triglyceride(TG), and Total Cholesterol(CHO), were analyzed using a reagent kit purchased from the Nanjing Jiancheng Bioengineering Institute.

## Glucose tolerance test (GTT) and insulin tolerance test (ITT)

GTT was performed after 16 weeks of HFD feeding, while ITT was conducted after 15 weeks of HFD feeding. After a 16 h fast, the mice underwent GTT and ITT assays. Mice were injected intraperitoneally with 1 g/kg glucose or 0.75 IU/kg insulin, and blood glucose concentrations were measured at the indicated time points (0, 15, 30, 60, and 120 min). The area under the curve (AUC) was then calculated using the conventional trapezoidal rule.

## Distribution of macrophages in vivo

Macrophages were fluorescently labeled with 1,1’-dioctadecyl-3,3,3’,3’-tetramethylindocarbocyanine dye (Beyotime, China) according to the manufacturer’s protocol and subsequently examined using a fluorescence microscope. To assess the in vivo distribution of macrophages, 1 × 10^6 Dil-labeled macrophages were intravenously injected into MASH mice. Macrophage distribution across various organs was visualized using small-animal in vivo imaging (Bruker Xtreme BI) and microscope (Olympus, Japan) at the indicated time points.

### H&E, IHC, DHE and ORO staining

H&E staining was performed by ServiceBio (Wuhan, China). IHC staining was conducted as previously described [26]. Lipid staining (Beyotime, China) and Dihydroethidium (DHE) staining (MCE, USA) were performed using a modified ORO staining kit according to the manufacturer’s instructions. All samples were observed under an inverted microscope (Olympus, Japan).

### siRNA and plasmid cell transfection

Cell transfection was performed as previously described [27], Plasmids and siRNA were separately purchased from Miaoling Biology (Wuhan, China) and RiBoBio (Guangzhou, China). Cells were transfected using Lipofectamine 2000 (Invitrogen, USA) following the manufacturer’s instructions. The siRNA sequences are listed in Supplementary Table S2.

### Primary hepatocyte isolation

Mouse primary hepatocytes were isolated using a standard two-step liver perfusion procedure. Briefly, livers were perfused with EGTA(MCE, USA) to loosen cell–cell junctions and subsequently digested with 0.05% collagenase(GIBCO, NY). The dissociated tissue was filtered through a 70-µm mesh, and hepatocytes were collected by repeated centrifugation at 70×g for 3 min.

### Isolation of primary liver macrophages

Mouse primary liver macrophages were isolated from the non-parenchymal cell fraction by Percoll density-gradient separation. Briefly, non-parenchymal cells were suspended in HBSS and loaded onto a two-step 25%–50% Percoll gradient. Following centrifugation at 200×g, cells recovered from the interphase were collected as the hepatic macrophage-enriched population. After washing to remove residual gradient medium, the cells were resuspended in DMEM containing 10% fetal bovine serum and used for further analysis.

### In vitro lipotoxic model

Palmitic acid (PA) and oleic acid (OA) stock solutions (20 mM) were pre-complexed with fatty acid-free bovine serum albumin (BSA) before use. Briefly, sodium palmitate and sodium oleate (Sigma-Aldrich, St. Louis, MO, USA) were each dissolved in 0.1 M NaOH at 70 °C, then mixed with 20% fatty acid-free BSA and incubated at 37 °C for 1 h to allow conjugation. Control BSA solution was prepared in parallel by mixing NaOH with fatty acid-free BSA at the same final concentrations and incubating under identical conditions. All solutions were sterilized by filtration through 0.22 μm filters and stored at -20 °C until use. To establish the in vitro lipotoxicity model, AML12 cells were treated with OA and PA at final concentrations of 500 µM and 250 µM, respectively (OA: PA = 2:1), for 48 h.

### Stable isotope tracing of fatty acid β-oxidation

Fatty acid β-oxidation was assessed by 13C-16 palmitate tracing coupled with targeted metabolomics. Briefly, 13C-16 palmitate was conjugated to fatty acid-free bovine serum albumin (BSA) and added to the culture medium. After 3 h of incubation, cells were washed with ice-cold PBS and intracellular metabolites were extracted with pre-cooled extraction solvent. Following centrifugation, supernatants were collected and analyzed by liquid chromatography–tandem mass spectrometry (LC-MS/MS) to determine the isotopologue distribution of palmitate-derived acyl-CoA species. The relative abundances of M + 16, M + 14, M + 12, M + 10, M + 8, M + 6, M + 4, and M + 2 isotopologues were quantified to evaluate exogenous palmitate utilization and mitochondrial β-oxidation activity.

### Metabolite extraction and LC-MS/MS analysis

Metabolites from FABP4-knockdown and control BMDMs were extracted with 1 mL of pre-cooled methanol/acetonitrile/water (v/v/v,2:2:1). Briefly, samples were sonicated in an ice bath for 1 h and then incubated at -20 °C for 1 h, followed by centrifugation at 14,000 g for 20 min at 4 °C. The supernatants were collected and transferred to sampling vials for LC-MS analysis. Protein concentrations were determined using a BCA assay. Metabolomic profiling was performed using a UPLC-ESI-Q-Orbitrap-MS system consisting of a Shimadzu Nexera X2 LC-30AD UHPLC system (Shimadzu, Japan) coupled to a Q-Exactive Plus mass spectrometer (Thermo Scientific, San Jose, CA, USA). For chromatographic separation, samples were analyzed on an ACQUITY UPLC® HSS T3 column (2.1 × 100 mm, 1.8 μm; Waters, Milford, MA, USA). Raw MS data were processed using MS-DIAL for peak alignment, retention time correction, and peak area extraction. Multivariate data analysis and modeling were performed using R (version 4.0.3) and relevant R packages.

### Immunoprecipitation-mass spectrometry (IP-MS)

His-tagged PPARα was overexpressed in BMDMs, purified using anti-His magnetic beads, and analyzed by mass spectrometry to identify associated proteins. Mass spectrometry was performed by Bioprofile (Shanghai, China), Immunoprecipitated protein complexes were subjected to proteolytic digestion, and peptide concentrations were determined prior to LC-MS/MS analysis. Peptides were separated using an Easy nLC 1200 nano-flow liquid chromatography system (Thermo Scientific) and analyzed on a Q-Exactive HF-X mass spectrometer (Thermo Scientific) operated in data-dependent acquisition (DDA) mode. Following each full MS scan, the top 20 most intense precursorions were selected for MS2 analysis. MS2 spectra were acquired at a resolution of 15,000 at m/z 200, with an AGC target of 1 × 10^5, a maximum injection time of 50 ms, an isolation window of 1.6 m/z, and a normalized collision energy (NCE) of 28 using HCD fragmentation. Raw data were searched using MaxQuant (version 2.4.14.0). Candidate PPARα-associated proteins were identified by comparing proteins detected in the IP sample with those in the negative control, and proteins showing presence or marked enrichment in the IP sample, together with peptide support, were considered candidate interactors. The mass spectrometry proteomics data have been deposited to the Proteomexchange Consortium via the PRIDE partner repository with the dataset identifier PXD077311.

### mRNA isolation and qPCR assay

Total RNA was extracted from cells and tissues using TRIzol reagent (Invitrogen, USA) and reverse transcribed into cDNA using ReverTra Ace qPCR RT Master Mix (Toyobo, Japan), following the manufacturer’s instructions. Quantitative PCR was performed using UltraSYBR mixture (Cwbio, China) on a Roche LightCycler 96 under standard PCR conditions. GAPDH was used as an internal control. The primers used are listed in Supplementary Table S3.

### Western blot analysis

Proteins were separated by SDS-PAGE and transferred onto membranes by electrophoresis. Immunoblotting and chemiluminescent detection were performed to analyze protein expression in equal amounts of each sample. For antibody details, see Supplementary Table S5.

### Immunofluorescence (IF) staining

Cells were collected and centrifuged onto coverslips, fixed with 4% paraformaldehyde for 30 min, and permeabilized with 0.2% Triton X-100 for 15 min at room temperature. Fixed cells were blocked with 5% BSA and incubated with the primary antibody in PBS at 4 °C overnight. The corresponding secondary antibody conjugated to FITC (Servicebio, China) or CY3 (Servicebio, China) was applied for 2 h. Nuclei were counterstained with 4′,6-diamidino-2-phenylindole (DAPI) (Servicebio, China). IF images were captured using a confocal microscope (Leica, Germany) or a fluorescence microscope (Olympus, Japan). For antibody details, see Supplementary Table S4.

### Nile red staining

Cells were fixed with 4% paraformaldehyde for 20 min at room temperature, then stained with Nile Red (1 µM in PBS; KKL, USA) for 10 min at room temperature. Lipid deposition was visualized and quantified by fluorescence microscopy.

#### Immunoprecipitation

BMDMs, RAW264.7, and HEK 293T cells were lysed in ice-cold lysis buffer supplemented with freshly added protease and phosphatase inhibitors (MCE, USA). After preclearing with protein A/G-agarose beads (Santa Cruz Biotechnology, USA), lysates were incubated with IgG, anti-FABP4, anti-Flag, or anti-PPARα antibody overnight at 4 °C with continuous inversion. Immunocomplexes were precipitated using protein A/G-agarose beads for approximately 3 h. The beads were then collected, and immunocomplexes were eluted by boiling in SDS loading buffer before immunoblot analysis with the indicated antibodies.

### Flow cytometry

Cultured cells were harvested from the liver or collected from culture dish and washed twice with cold PBS. To block Fc receptors, the cells were pre-incubated with anti-mouse CD16/32 antibodies (1.0 µg in 100 µL) for 10 min on ice. The cells were then stained with specific antibodies for 30 min at 4 °C in the dark, washed three times with cold PBS, and analyzed by flow cytometry. Antibody information is provided in Supplementary Table S5.

#### ChIP-qPCR

ChIP-qPCR was performed using the Magnify Chromatin Immunoprecipitation System (ABclonal, China) according to the manufacturer’s protocol. Briefly, cells were collected and fixed, and the chromatin was cross-linked, extracted, and sheared. Immunoprecipitation was performed using anti-STAT1 (Cell Signaling Technology, USA). The primer sequences used for ChIP-qPCR were as follows: Murine fabp4, GATGCCCTGACCATGTGACT (sense) and TTGCCAGGGAGAACCAAAGT (antisense). Results were obtained from three independent experiments.

### Statistical analysis

All experiments were performed at least three times. Results are expressed as the mean ± SD. Sample sizes were determined based on our preliminary experiments, previous studies, and standard practice in similar animal and cell-based experiments, although a formal power analysis was not performed. Student’s *t*-test (two-tailed) was used for comparisons between two groups, while one-way analysis of variance with Bonferroni adjustment was applied for multiple comparisons. Correlation analyses were conducted using Pearson’s correlation test. Statistical significance was set at *p* < 0.05. Analyses were performed using GraphPad v9.5 software.

## Results

### Fibrin deposition surrounding proinflammatory macrophages in MASH livers

**Fig. 1:**
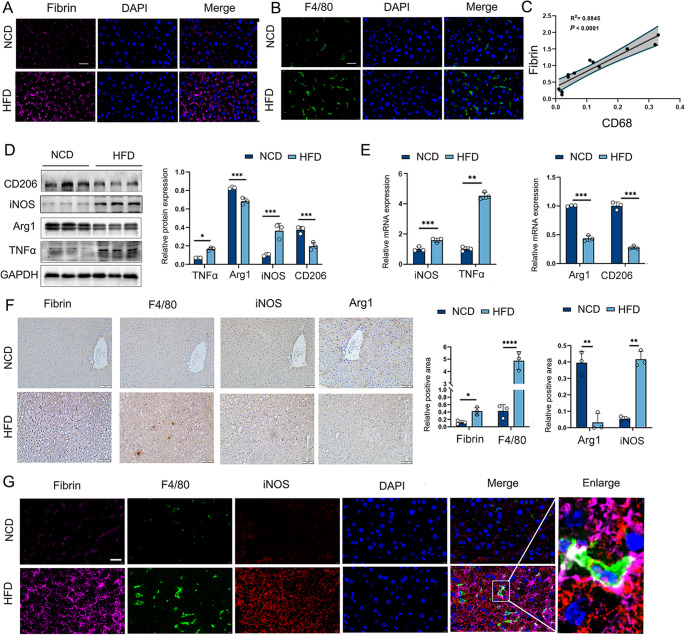
Fibrin deposition surrounding proinflammatory macrophages in MASH livers. (**A**) Representative immunofluorescence images of liver sections from HFD-fed and NCD-fed mice for fibrin (Cy5) (scale bar, 50 μm). (**B**) Representative immunofluorescence images of liver sections from HFD-fed and NCD-fed mice stained with FITC-conjugated anti-F4/80 antibodies (scale bar, 50 μm). (**C**) Correlation of fibrin and CD68 expression in liver tissue from patients with MASH detected by immunofluorescence(*n* = 12). (**D**) Protein levels in the livers of NCD-fed mice and HFD-fed mice with MASH. (**E**) mRNA levels in the livers of NCD-fed mice and HFD-fed mice with MASH. (**F**) Representative immunohistochemical images of liver sections from NCD-fed mice and HFD-fed mice with MASH stained for fibrin, F4/80, iNOS, and Arg1 (*n* = 3, scale bar, 50 μm). (**G**) Representative immunofluorescence images of liver sections from NCD-fed mice and HFD-fed mice with MASH stained for fibrin (Cy5), F4/80 (FITC), and iNOS (Cy3) (scale bar, 50 μm). Data are presented as mean ± SD. In all scatter plots with bars, each data point represents one individual. *p < 0.05, **p < 0.01, ***p < 0.001, ****p < 0.0001

We examined fibrin and macrophage expression in liver biopsies from patients with MASH and HFD-fed MASH mice. Immunofluorescence analysis revealed a significant increase in fibrin deposition and macrophage content in the livers of HFD-fed MASH mice and patients with MASH (Fig. 1A, B; Fig. S1A, B). Fibrin expression was positively correlated with macrophage count in clinical samples (Fig. 1C). We then assessed inflammation-related markers in mouse liver tissue at both the protein (Fig. 1D) and mRNA (Fig. 1E) levels. CD206 and Arg1 levels were significantly lower in HFD-fed MASH mice than in normal chow diet (NCD) mice, whereas TNFα and iNOS levels were higher in HFD-fed MASH mice than that in NCD mice. Additionally, immunohistochemical analysis showed fibrin content and macrophage populations increased in HFD-fed MASH mice. Specifically, these mice exhibited numerous iNOS⁺ proinflammatory macrophages and fewer Arg1⁺ anti-inflammatory macrophages (Fig. 1F). Multicolor immunofluorescence analysis of mouse liver and clinical samples tissue further demonstrated an increase in iNOS-positive proinflammatory macrophages surrounding fibrin deposits (Fig. 1G; Fig. S1C). Collectively, these findings suggest that fibrin deposition may contribute to the accumulation of proinflammatory macrophages in the livers of patients with MASH and HFD-fed MASH mice.

### Macrophages adopt an anti-inflammatory phenotype in a soft fibrin matrix

**Fig. 2:**
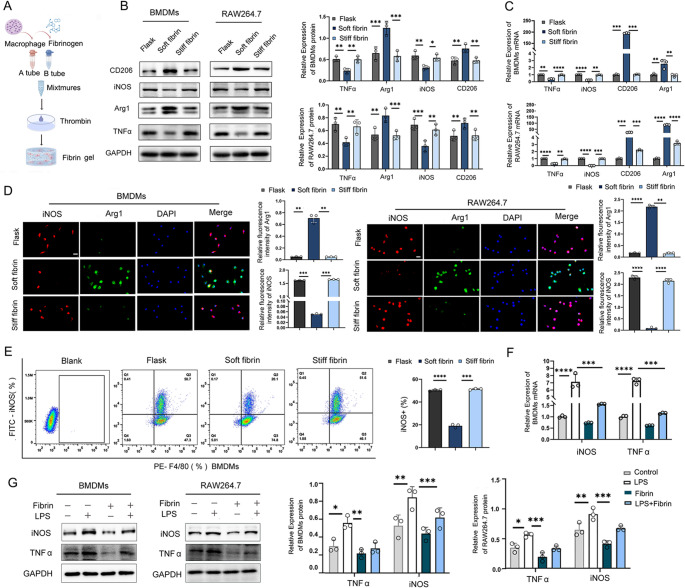
Macrophages adopt an anti-inflammatory phenotype in a soft fibrin matrix. (**A**) Fibrin gels for three-dimensional (3D) macrophage culture. (**B**) Effect of matrix stiffness on macrophage polarization in BMDMs and RAW264.7 cells at the protein level. (**C**) Effect of matrix stiffness on macrophage polarization in BMDMs and RAW264.7 cells at the mRNA level. (**D**) Representative immunofluorescence images of BMDMs and RAW264.7 cells stained for iNOS (Cy3) and Arg1 (FITC) (scale bar, 50 μm). (**E**) Proportions of iNOS⁺ BMDMs analyzed by flow cytometry. (**F**) mRNA levels of TNFα and iNOS in LPS-stimulated BMDMs cultured in soft fibrin hydrogels. (**G**) Protein levels of TNFα and iNOS in soft fibrin hydrogels containing LPS-stimulated BMDMs and RAW264.7 cells. Data are presented as mean ± SD. In all scatter plots with bars, each data point represents one independent experiment. *p < 0.05, **p < 0.01, ***p < 0.001, ****p < 0.0001

Fibrin is an important component of extracellular matrices, cells can sense and respond to mechanical forces within their microenvironment [28]. A previous study demonstrated fibrin stiffness influences tumor biological behavior through mechanical signaling [29]. Here, we investigated the effects of fibrin stiffness on macrophage polarization. Macrophages were seeded in fibrin matrix or flasks. Figure 2A depicts a three-dimensional (3D) culture system in which macrophages were grown in a fibrin matrix. Western blot (Fig. 2B) and RT-PCR (Fig. 2C) revealed that iNOS and TNFα levels decreased, whereas Arg1 and CD206 levels increased when macrophages were cultured in a soft fibrin matrix compared to those grown in flasks or stiff fibrin matrix. Immunofluorescence analysis further showed that BMDMs and RAW264.7 cells cultured in soft fibrin exhibited reduced iNOS expression but increased Arg1 expression compared with those grown in conventional flasks or stiff fibrin (Fig. 2D). Flow cytometry yielded consistent findings (Fig. 2E; Fig. S2A). To examine the impact of soft fibrin on proinflammatory macrophages, we measured TNFα and iNOS in LPS-stimulated cells, Both markers were reduced at the mRNA and protein levels in the soft fibrin hydrogel (Fig. 2F, G; Fig. S2B). Collectively, these findings indicate that a soft fibrin matrix promotes anti-inflammatory macrophage polarization, whereas a stiff fibrin matrix induces a proinflammatory phenotype.

### Macrophages seeded in a soft fibrin matrix alleviated obesity and insulin resistance in high-fat diet-induced MASH mice

**Fig. 3:**
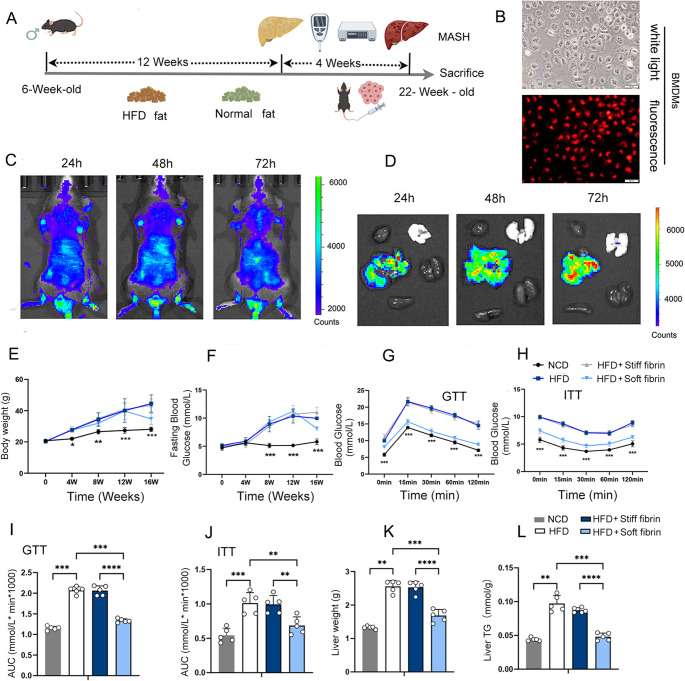
Macrophages seeded in a soft fibrin matrix alleviated obesity and insulin resistance in high-fat diet-induced MASH mice. (**A**) Schematic depiction of the in vivo experiments evaluating the effects of BMDMs seeded in soft or stiff fibrin in HFD-fed MASH mice. (**B**) Dil-labeled BMDMs. **C**. Fluorescence-labeled BMDMs are injected intravenously, and optical in vivo imaging is performed at 24, 48, and 72 h post-injection. (**D**) Fluorescence intensity in specified organs is imaged at 24, 48, and 72 h post-injection. (**E**) Body weight (*n* = 5). (**F**) Fasting blood glucose levels (*n* = 5). (**G−J**) Glucose tolerance test (GTT) and insulin tolerance test (ITT) results, along with the corresponding area under the curve (AUC) at the indicated time points in the NCD, HFD, stiff fibrin groups and soft fibrin groups (*n* = 5). (**K**) Liver weight(*n* = 5). **L**. triglyceride (TG) levels in different groups (*n* = 5). Data are presented as mean ± SD. In all scatter plots with bars, each data point represents one individual. *p < 0.05, **p < 0.01, ***p < 0.001, ****p < 0.0001

To further investigate the role of macrophages seeded in a soft fibrin matrix in the pathophysiology of MASH in vivo, an animal experiment was performed (Fig. 3A). A total of 1 × 10^6 BMDMs or RAW264.7 macrophages cultured in fibrin gels of varying stiffness were recovered, resuspended in 100 µL PBS, and injected via the tail vein once weekly for four weeks. Before injection, trypan blue exclusion and flow cytometry confirmed > 90% viability and > 95% macrophage purity, respectively (Fig. S3A, B). Three days after the final injection, mice were euthanized for further analysis. To assess macrophage distribution, Dil-labeled macrophages BMDMs and RAW264.7 (Fig. 3B; Fig. S3C) were injected into the tail veins of MASH mice. Fluorescence imaging revealed that macrophages primarily localized to the liver (Fig. 3C, D; Fig. S3D, E). Similarly, fluorescence microscopy performed 48 h after tail vein injection showed that BMDMs were predominantly located in the liver, with only a small fraction detected in the spleen and minimal presence in the heart, lungs, and kidneys (Fig. S3F). These results confirm that macrophages administered through the tail vein mainly home to the liver. To further assess the persistence and polarization state of transferred macrophages in vivo, soft fibrin-cultured macrophages were labeled with Dil prior to tail-vein injection, and immunofluorescence staining of liver tissues was performed 72 h after transfer. Dil-labeled macrophages were positive for CD206, suggesting that these cells retained, at least in part, an M2-like phenotype after entering the liver (Fig. S3G).

Subsequently, we assessed several other indicators in the mice. The body weight did not increase further in mice injected with BMDMs cultured in a soft fibrin matrix, whereas no such effect was observed in those receiving macrophages cultured in a stiff fibrin matrix (Fig. 3E). Fasting blood glucose levels (Fig. 3F) as well as glucose and insulin tolerance improved in the HFD+soft fibrin group compared with both HFD-fed MASH mice and the HFD+stiff fibrin group mice (Fig. 3G–J). In addition, liver weight and triglyceride (TG) levels were lower in the HFD+soft fibrin group than in both HFD and the HFD+stiff fibrin group mice (Fig. 3K, L and Fig. S3H). These findings indicate that macrophages seeded in a soft fibrin matrix alleviated obesity and insulin resistance in high-fat diet-induced MASH mice.

### Macrophages seeded in a soft fibrin matrix alleviated hepatic steatosis, inflammation and oxidative stress in vitro and in vivo

**Fig. 4:**
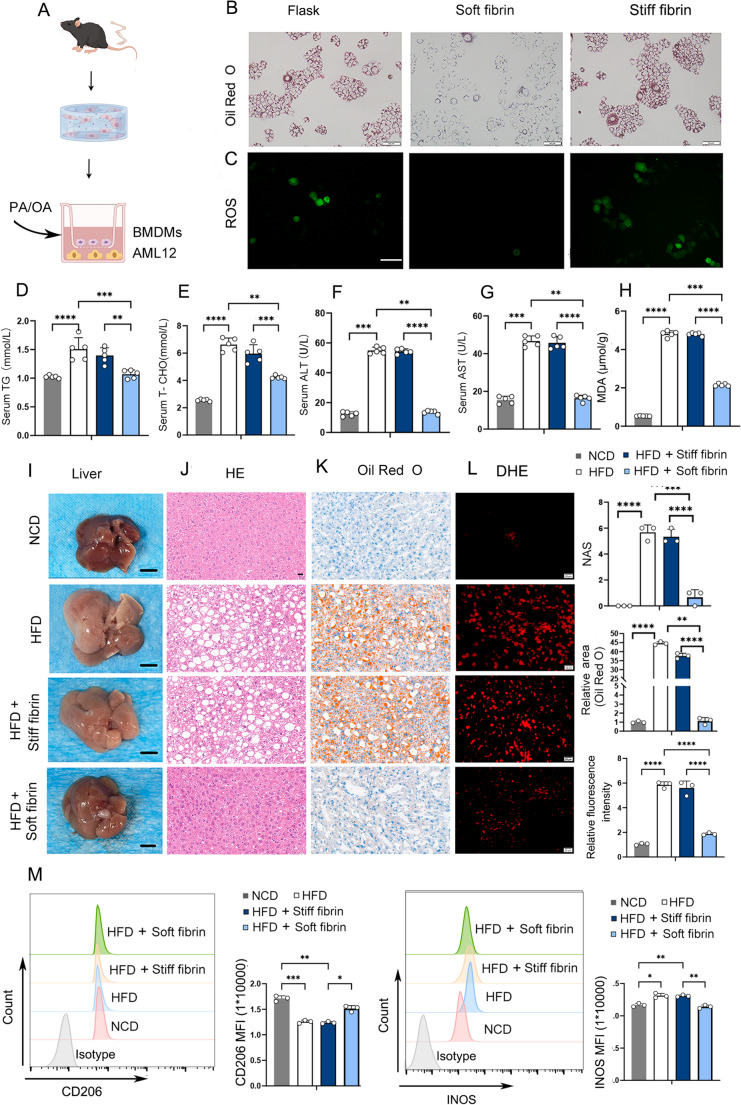
Macrophages seeded in a soft fibrin matrix alleviated hepatic steatosis, inflammation and oxidative stress in vitro and in vivo. (**A**) Schematic representation of the co-culture of AML12 hepatocytes and BMDMs. (**B**) Oil Red O (ORO) staining in AML12 cells after co-culture with BMDMs seeded in fibrin matrices of different stiffness for 48 h. (**C)** ROS detection of AML12 cells after co-culture with BMDMs seeded in different stiffness fibrin matrices for 48 h. (**D**) Serum TG levels (*n* = 5), (**E**) Serum total cholesterol (TC) levels (*n* = 5), **F**. Serum alanine aminotransferase (ALT) levels (*n* = 5), (**G**) Serum aspartate aminotransferase (AST) levels (*n* = 5) and (**H**) Hepatic malondialdehyde (*n* = 5). (**I**) Gross images of liver tissues. (**J**) H&E staining(*n* = 3). (**K**) ORO staining(*n* = 3) and (**L**) Dihydroethidium (DHE) staining (*n* = 3, scale bar, 20 μm). (**M**) Hepatic macrophages are isolated from the liver using gradient centrifugation. The proportions of iNOS+ macrophages and CD206 + macrophages are analyzed via flow cytometry with the respective antibodies(*n* = 3). Data are presented as mean ± SD. In all scatter plots with bars, each data point represents one individual. *p < 0.05, **p < 0.01, ***p < 0.001, ****p < 0.0001

To determine whether macrophage seeding in a fibrin matrix affects hepatic steatosis and inflammation under metabolic stress, bone marrow-derived macrophages were seeded in flasks, soft fibrin matrices, and stiff fibrin matrices for 48 h, followed by co-culture with AML12 hepatocytes. In the co-culture experiments, AML12 hepatocytes were seeded in the lower chamber of a 6-well Transwell plate at approximately 4 × 10^5 cells/well and cultured for 24 h before co-culture. BMDMs were cultured in fibrin gels of different stiffness for 48 h, and after digestion and collection, macrophages were seeded into the upper chamber of a 0.4 μm Transwell insert at approximately 1 × 10^6 cells/well. The cells were then stimulated with palmitic acid (PA) and oleic acid (OA) for 48 h under serum-free conditions (Fig. 4A). Under these conditions, hepatocytes co-cultured with macrophages in flasks and stiff fibrin matrices showed a greater increase in lipid droplet accumulation and higher levels of ROS than those in soft fibrin matrices (Fig. 4B, C).

Similarly, mice in the HFD+soft fibrin group exhibited lower levels of serum lipids and liver enzymes compared with both HFD and the HFD+stiff fibrin group mice (Fig. 4D-G; Fig. S4A-C). Furthermore, malondialdehyde (MDA) levels in liver tissue were also reduced in the HFD+soft fibrin group (Fig. 4H). Then, inflammation, lipid deposition and oxidative stress in the liver were compared across groups based on liver morphology (Fig. 4I), hematoxylin and eosin (H&E) staining (Fig. 4J and Fig. S4D), oil red O (ORO) staining (Fig. 4K; Fig. S4E) and Dihydroethidium (DHE) staining (Fig. 4L). Hepatic macrophage function was further assessed in vivo using flow cytometry, which revealed that the HFD+soft fibrin group had significantly fewer iNOS⁺ macrophages and more CD206⁺ macrophages in the liver (Fig. 4M). Similarly, the downregulation of CD206 and Arg1 in the liver of MASH mice was successfully reversed by soft fibrin-cultured BMDMs. Notably, TNFα and iNOS protein levels, which were upregulated in the liver tissue of MASH mice, were significantly reduced by soft fibrin-cultured macrophages (Fig. S4F). Immunofluorescence analysis revealed that IL-1β expression was reduced in soft fibrin-cultured macrophages and was negatively correlated with CD163 expression, a marker of M2 macrophages (Fig. S4G). Collectively, these findings indicate that macrophages seeded in a soft fibrin matrix mitigate hepatic steatosis, inflammation and oxidative stress. While ameliorating the macrophage-mediated immune microenvironment in the liver.

### Soft fibrin modulates the anti-inflammatory polarization of macrophages through FABP4

**Fig. 5:**
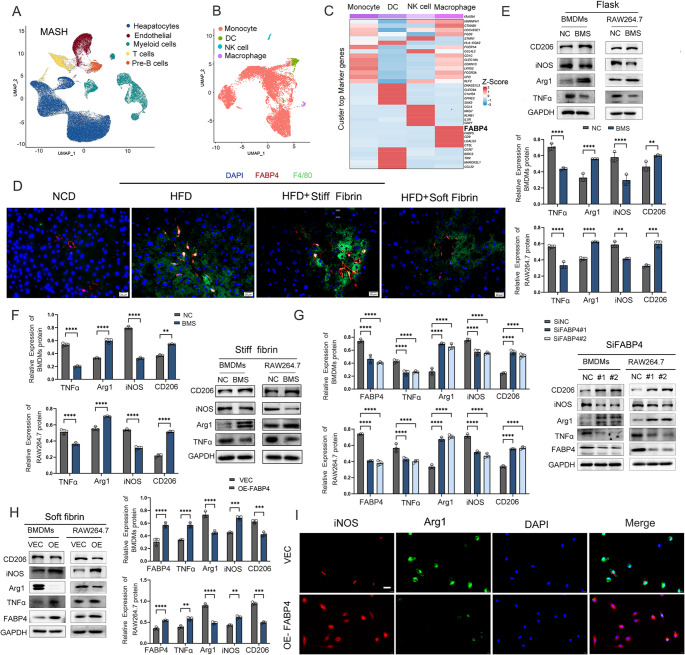
Soft fibrin modulates the anti-inflammatory polarization of macrophages through FABP4. (**A**) Five subpopulations of cells identified based on gene expression profiles. (**B**) Myeloid cells subjected to secondary clustering. (**C**) Heatmap of the top marker genes following secondary clustering. (**D**) Representative immunofluorescence staining of liver sections from mice treated with anti-FABP4 and anti-F4/80 antibodies (scale bar, 20 μm). (**E**) Protein expression associated with macrophage polarization in BMDMs and RAW264.7 cells treated with an FABP4 inhibitor BMS. (**F**) Protein expression associated with macrophage polarization in BMDMs and RAW264.7 cells seeded in stiff fibrin and treated with an FABP4 inhibitor BMS. (**G**) Protein expression associated with macrophage polarization in BMDMs and RAW264.7 cells seeded in stiff fibrin following FABP4 knockdown. (**H**) Protein expression associated with macrophage polarization in BMDMs and RAW264.7 cells seeded in soft fibrin following FABP4 overexpression. (**I**) Representative immunofluorescence images of BMDMs overexpressing FABP4 using Arg1 (FITC) and iNOS (Cy3) antibodies (scale bar, 20 μm). Data are presented as mean ± SD. In all scatter plots with bars, each data point represents one independent experiment. *p < 0.05, **p < 0.01, ***p < 0.001, ****p < 0.0001

To decipher the molecular mechanisms by which macrophages seeded in soft fibrin promote anti-inflammatory polarization, we performed single-cell analysis using human MASH datasets GSE202379 and GSE169446. Briefly, raw count matrices were downloaded from GEO and imported into Seurat to generate dataset-specific objects (for both GSE202379 and GSE169446, min.cells = 3 and min.features = 200). The two datasets were then merged, and sample metadata were incorporated for subsequent analyses. The merged object was normalized using the LogNormalize method, followed by data scaling. Next, 2,500 highly variable genes were selected, and principal component analysis was performed. The top 10 principal components were then used for neighborhood graph construction, clustering, and UMAP visualization. Cell identities were assigned based on ScType, SingleR, and cluster-specific features, and were further consolidated into the major cell populations presented in the manuscript (Fig. 5A). After the global clustering and annotation, myeloid cells were extracted for secondary clustering analysis, which identified four myeloid subpopulations(Fig. 5B). FABP4 and FABP5 were highly expressed in macrophages and were among the signature genes of these cells (Fig. 5C). Since FABP5 primarily plays a role in skin inflammation and FABP4 is highly expressed in adipocytes and macrophages [19], we focused on FABP4 for further investigation. Immunofluorescence results suggest that macrophages in HFD group mice expressed higher levels of FABP4 in the liver than NCD group mice, and after treatment with soft fibrin matrix-cultured macrophages, FABP4 expression in hepatic macrophages was significantly reduced in MASH mice (Fig. 5D). Immunohistochemistry and western blotting yielded consistent findings (Fig. S5A, B).

We then examined the effect of fibrin stiffness on FABP4 expression in macrophages. Macrophages cultured in soft fibrin exhibited lower FABP4 protein expression than those cultured in stiff fibrin or flasks (Fig. S5C). It remains to be determined whether FABP4 plays a critical role in macrophage polarization. We found that in macrophages cultured in stiff fibrin or flasks, FABP4 inhibition using BMS309403 (BMS) significantly decreased iNOS and TNFα protein levels while increasing Arg1 and CD206 protein expression (Fig. 5E, F). Furthermore, in macrophages cultured in a stiff fibrin matrix, knockdown of FABP4 reduces the levels of iNOS and TNFα, while increasing the levels of CD206 and Arg1, which are already decreased (Fig. 5G). However, macrophages cultured in soft fibrin, FABP4 overexpression increased iNOS and TNFα expression while decreasing Arg1 and CD206 levels (Fig. 5H). Immunofluorescence analysis confirmed these findings (Fig. 5I and Fig. S5D). These data indicate that FABP4 plays a crucial role in fibrin-induced macrophage polarization and FABP4 promotes the pro-inflammatory M1 polarization of macrophages.

### Inhibition of the ITGB2/STAT1 axis reduces FABP4 transcription and promotes anti-inflammatory macrophage polarization

**Fig. 6:**
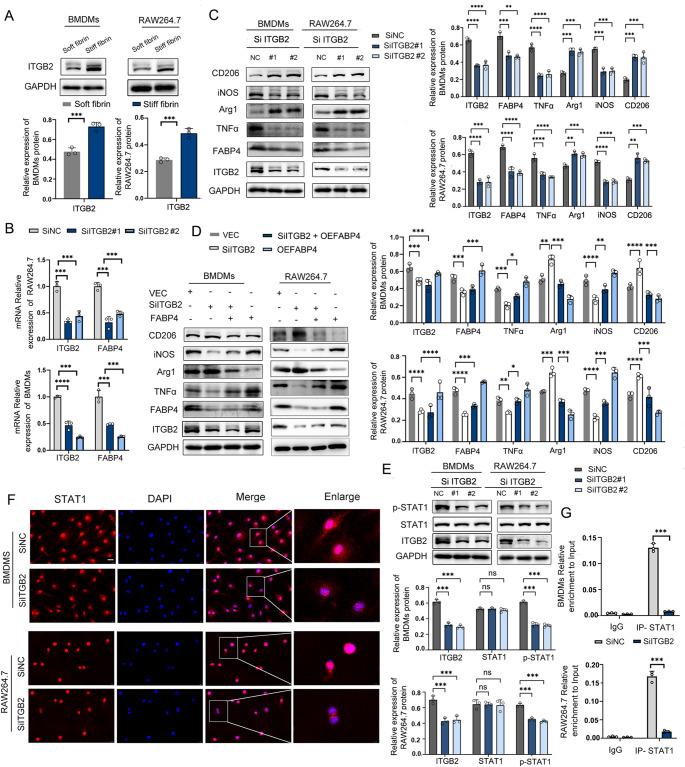
Inhibition of the ITGB2/STAT1 axis reduces FABP4 transcription and promotes anti-inflammatory macrophage polarization. (**A**) ITGB2 levels in BMDMs and RAW264.7 cells cultured on soft and stiff fibrin gels, analyzed using western blotting. (**B**) FABP4 mRNA expression in BMDMs and RAW264.7 cells after ITGB2 silencing. (**C**) Western blotting analysis of macrophage polarization-associated protein expression in BMDMs and RAW264.7 cells after ITGB2 silencing. (**D**) Western blotting analysis of macrophage polarization protein expression after rescue experiments. (**E**) Western blotting analysis of STAT1 pathway protein expression after ITGB2 silencing. (**F**) Representative immunofluorescence (IF) staining of STAT1 in BMDMs and RAW264.7 cells after ITGB2 silencing (scale bar, 20 μm). (**G**) BMDMs and RAW264.7 cells were treated with ITGB2 siRNA for 48 h, followed by chromatin immunoprecipitation-qPCR (ChIP-qPCR) using an anti-STAT1 antibody and specific primers for FABP4. Data are presented as mean ± SD. In all scatter plots with bars, each data point represents one independent experiment. *p < 0.05, **p < 0.01, ***p < 0.001, ****p < 0.0001

Next, we sought to elucidate the mechanism underlying the activation of FABP4 in fibrin-induced macrophage polarization. Given that β integrins (ITGB) can mediate mechanotransduction, we assessed the expression of β subunits (β1–β8) and found that β2 integrin (ITGB2) expression was markedly increased in macrophages cultured in stiff matrices (Fig. 6A; Fig. S6). We hypothesized that fibrin matrix stiffness regulates macrophage polarization through ITGB2-dependent mechanotransduction pathway, which subsequently affects downstream signaling and FABP4-associated phenotypic remodeling. To determine the functional role of ITGB2, we next silenced ITGB2 in macrophages using interfering RNA. ITGB2 knockdown significantly decreased FABP4 expression at both the mRNA and protein levels and was accompanied by a shift toward an anti-inflammatory macrophage phenotype, as evidenced by reduced iNOS and TNFα expression together with increased Arg1 and CD206 expression (Fig. 6B, C). We then asked whether the effect of ITGB2 on macrophage polarization was mediated through FABP4. To address this question, we performed a rescue experiment by manipulating FABP4 in the context of ITGB2 knockdown. The results showed that restoration of FABP4 partially reversed the phenotypic changes induced by ITGB2 silencing (Fig. 6D), supporting the notion that FABP4 functions downstream of ITGB2 in this process. These results support the conclusion that ITGB2 contributes, at least in part, to fibrin matrix-induced FABP4 activation and pro-inflammatory macrophage polarization.

Previous studies have reported that signal transducer and activator of transcription 1 (STAT1) mediates mechanotransduction and plays a key role in macrophage polarization [27, 30–31]. Therefore, we investigated whether ITGB2 expression modulates STAT1 phosphorylation in macrophages. ITGB2 silencing reduced phosphorylated STAT1 (p-STAT1) activation in macrophages (Fig. 6E). Given that STAT1 activation is mediated through phosphorylation, which promotes its nuclear translocation, we further verified this mechanism by immunofluorescence assay (Fig. 6F). To determine whether STAT1 directly influences FABP4 expression, we analyzed STAT1 binding to the FABP4 promoter using chromatin immunoprecipitation followed by quantitative PCR (ChIP-qPCR). STAT1 binding was significantly reduced in ITGB2-silenced macrophages compared to control cells (Fig. 6G). Taken together, these findings support a model in which ITGB2 senses fibrin matrix stiffness and promotes STAT1 activation, thereby enhancing FABP4 transcription and driving pro-inflammatory macrophage polarization. Conversely, ITGB2 inhibition attenuates STAT1 activation, reduces FABP4 expression, and promotes anti-inflammatory macrophage polarization.

### FABP4 promotes anti-inflammatory polarization of macrophages by regulating lipid metabolism

**Fig. 7:**
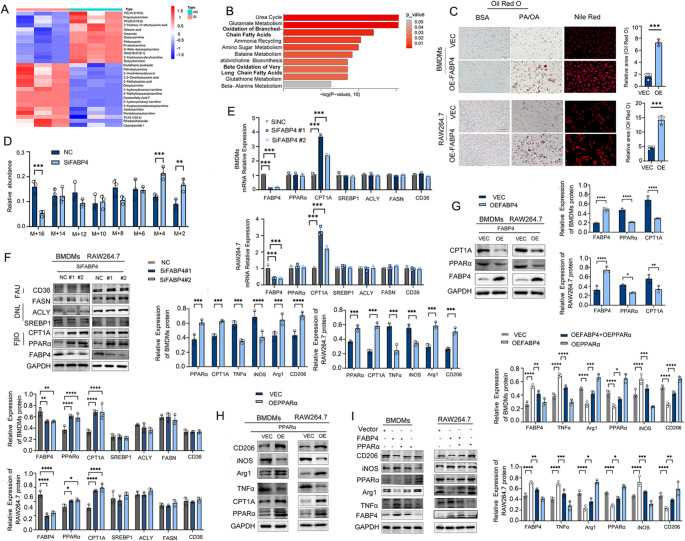
FABP4 promotes anti-inflammatory polarization of macrophages by regulating lipid metabolism. (**A**) Differentially expressed fatty acid metabolites (*p* < 0.05) between the control and FABP4 knockdown groups of BMDMs seeded in stiff fibrin. (**B**) KEGG enrichment analysis of metabolite data showing dominant biological pathways associated with FABP4 knockdown compared with the control group. (**C**) Representative Oil Red O and Nile Red staining of BMDMs and RAW264.7 treated with OA/PA in FABP4-overexpression and control groups (scale bar, 20 μm). (**D**) ^13^C-16-palmitate tracing coupled with targeted metabolomics showing increased incorporation of labeled palmitate into downstream acyl-CoA intermediates and elevated palmitate-derived isotopologues in FABP4-silenced macrophages compared with control cells. (**E**) mRNA expression of lipid metabolism-related genes in BMDMs and RAW264.7 cells following FABP4 knockdown for 48 h. (**F**) Western blotting analysis of FABP4 and fatty acid metabolism-related proteins following FABP4 knockdown for 48 h. (**G**) Western blotting analysis of PPARα and CPT1A in BMDMs and RAW264.7 cells treated with vector or FABP4-overexpressing plasmids for 48 h. (**H**) Protein expression of macrophage polarization markers in BMDMs and RAW264.7 cells treated with vector or PPARα-overexpressing plasmids for 48 h. **(I)** Western blotting analysis of macrophage polarization protein expression after the after rescue experiments. Data are presented as mean ± SD. In all scatter plots with bars, each data pointrepresents one independent experiment.^*^p < 0.05,^**^p < 0.01,^***^p < 0.001,^****^p < 0.0001

To investigate how FABP4 regulates macrophage polarization, we conducted the following experiment. Since FABP4 plays a key role in various metabolic disorders [32, 33], we performed non-targeted metabolomics in stiff fibrin-cultured BMDMs derived from FABP4-knockdown and wild-type cells. The results revealed a significant difference in fatty acid metabolites between the two groups (Fig. 7A). KEGG pathway enrichment analysis showed that proteins interacting with key genes were enriched in fatty acid β-oxidation (FβO) (Fig. 7B). Several studies have shown that macrophages absorb lipids and generate pro-inflammatory foamy macrophages both in vitro and in vivo [13]. To determine whether FABP4 regulates lipid metabolism in macrophages, we used PA/OA to induce a high-fat load and compared the lipid content between FABP4-overexpressing and control cells. Lipid droplet content was analyzed using Oil Red O staining (ORO) and Nile red staining, which revealed a significant increase in lipid droplets in FABP4-overexpressing cells compared to controls (Fig. 7C). We then compared lipid content in macrophages cultured in soft and stiff fibrin, as well as in flasks. Lipid droplet content was significantly lower in macrophages cultured in soft fibrin than in those cultured in stiff fibrin or flasks (Fig. S7A, B). A similar triglyceride profile was observed in macrophage lysates under these conditions (Fig. S7C, D). These results suggest that FABP4 regulates lipid metabolism in macrophages. To determine whether FABP4 affects fatty acid β-oxidation in macrophages, we performed ^13^C-16-palmitate tracing coupled with targeted metabolomics. Compared with control cells, FABP4-silenced macrophages showed increased incorporation of ^13^C-labeled palmitate into downstream acyl-CoA intermediates, with elevated abundances of multiple palmitate-derived isotopologues (Fig. 7D).

Next, we examined the relationship between FABP4 and lipid metabolism by assessing changes in key molecules associated with fatty acid uptake (FAU), de novo lipogenesis (DNL), and FβO at both the mRNA and protein levels. DNL-related genes (FASN, SREBP1, and ACLY) and the FAU-related gene CD36 showed no significant changes in expression at either level. However, the FβO-related molecules CPT1A and peroxisome proliferator-activated receptor alpha (PPARα) exhibited substantial alterations and were negatively correlated with FABP4 expression at the protein level (Fig. 7E-G). These findings suggest that FABP4 knockdown in macrophages enhances FβO, thereby promoting anti-inflammatory polarization. To test this hypothesis, we overexpressed PPARα and analyzed macrophage polarization. The results showed that PPARα overexpression decreased iNOS and TNFα expression while increasing Arg1 and CD206 levels compared to control cells (Fig. 7H). To determine whether PPARα contributes to the macrophage phenotype induced by the soft fibrin matrix, we inhibited PPARα in macrophages cultured under soft fibrin conditions in BMDMs. The soft fibrin matrix increased CPT1A expression, whereas PPARα inhibition attenuated this effect. In parallel, inhibition of PPARα also altered the polarization changes induced by the soft matrix (Fig. S7E). These findings indicate that PPARα is involved in the regulation of fatty acid oxidation and macrophage polarization in response to the soft fibrin matrix. Furthermore, to determine whether FABP4 regulates macrophage polarization via PPARα, we performed a rescue experiment. Interestingly, re-expression of FABP4 partially reversed the anti-inflammatory polarization induced by PPARα overexpression (Fig. 7I). These findings indicate that FABP4 knockdown promotes anti-inflammatory macrophage polarization by accelerating fatty acid β-oxidation.

### FABP4 competes with ubiquitin-specific peptidase 53 (USP53) to bind PPARα, maintaining PPARα stability

**Fig. 8:**
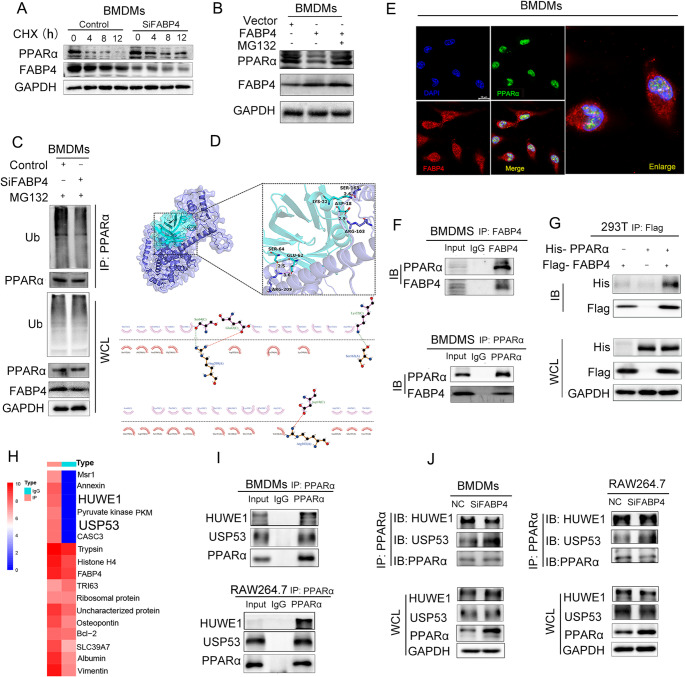
FABP4 competes with ubiquitin-specific peptidase 53 (USP53) for binding to PPARα, maintaining PPARα stability. (**A**) PPARα protein levels in BMDMs with FABP4 knockdown following CHX (100 µg/mL) treatment for the indicated times. (**B**) Western blotting analysis of PPARα expression after transient FABP4 overexpression and treatment with MG132 (10 µmol/L, 6 h). (**C**) Immunoblot analysis of PPARα ubiquitination in BMDMs transfected with siRNA. Macrophages were treated with MG132 for 6 h before immunoprecipitation. (**D**) Predicted protein–protein complex conformation between FABP4 and PPARα, determined using PyMol (v 2.2.0) and LigPlot+ (v 2.2.4). (**E**) Representative confocal microscopy images of FABP4 and PPARα expression in BMDMs, the showing colocalization of PPARα (FITC) and FABP4 (CY3) (scale bar, 10 μm). (**F**) Co-immunoprecipitation (Co-IP) analysis of endogenous FABP4 and PPARα interactions in BMDMs. (**G**) Co-IP analysis of exogenous FABP4 and PPARα interactions in HEK293T cells. (**H**) Heatmap of differentially expressed PPARα-interacting proteins in BMDMs identified by immunoprecipitation mass spectrometry (IP-MS). (**I**) Co-IP analysis of endogenous PPARα interactions with USP53 and HUWE1 in BMDMs and RAW264.7 cells, with IgG as a negative control. (**J**) Co-IP analysis of endogenous PPARα interactions with USP53 and HUWE1, showing that FABP4 knockdown enhances the interaction between USP53 and PPARα

Next, we explore the potential mechanisms underlying FABP4 regulation of PPARα activity. Based on the observations above, we found that interference with FABP4 expression did not affect PPARα mRNA levels but significantly increased PPARα protein levels. These findings suggest that FABP4 regulates PPARα protein at the post-transcriptional level. In fact, post-transcriptional regulation of PPARα protein has been identified as a key mechanism in previous studies [34]. Therefore, we further analyzed whether FABP4 affected the half-life of PPARα protein. FABP4 knockdown in macrophages cultured in stiff fibrin markedly reduced the degradation rate of newly synthesized PPARα protein in the presence of cycloheximide (CHX, 100 µg/mL) (Fig. 8A and Fig. S8A). Furthermore, in cells overexpressing FABP4, treatment with the proteasome inhibitor MG132 substantially restored PPARα protein levels (Fig. 8B and Fig. S8B). Additionally, ubiquitination levels of PPARα decreased upon FABP4 silencing in macrophages cultured in stiff fibrin (Fig. 8C and Fig. S8C). Given that FABP4 modulates PPARα function by regulating its degradation, we hypothesized that FABP4 physically interacts with PPARα. Consistent with this hypothesis, protein–protein docking analysis using PyMol (V.2.2.0) and GRAMM predicted a possible interaction between FABP4 and PPARα (Fig. 8D). immunofluorescence staining demonstrated that endogenous FABP4 colocalized with PPARα in macrophages (Fig. 8E and Fig. S8D). Consistently, Co-immunoprecipitation experiments in BMDMs, RAW264.7, and HEK293T cells confirmed that FABP4 associates with PPARα (Fig. 8F, G and Fig. S8E).

The ubiquitin-proteasome system is crucial for regulating protein stability. To identify the E3 ubiquitin ligase or deubiquitinase involved in PPARα polyubiquitination, we performed immunoprecipitation mass spectrometry (IP-MS). His-tagged PPARα was overexpressed in BMDMs, purified using anti-His magnetic beads, and analyzed by mass spectrometry to identify associated proteins. Among the identified E3 ubiquitin ligase or deubiquitinase, HUWE1 and USP53 were the most abundant (Fig. 8H). Co-immunoprecipitation assays confirmed that endogenous USP53 and HUWE1 interacted with PPARα (Fig. 8I). Because these findings suggest that FABP4-induced ubiquitination of PPARα is mediated by ubiquitin enzymes, we hypothesized that FABP4 may regulate PPARα ubiquitination by modulating the access of ubiquitin-related enzymes, particularly USP53, to PPARα. Supporting this hypothesis, neither USP53 nor HUWE1 protein levels were altered upon FABP4 knockdown. However, FABP4 knockdown increased the interaction between endogenous USP53 and PPARα in macrophages, whereas HUWE1 binding remained unchanged (Fig. 8J). These findings indicate that FABP4 enhances PPARα ubiquitination by disrupting the interaction between PPARα and USP53. To further investigate the mechanism by which USP53 regulates PPARα, we assessed the ubiquitination status of PPARα following USP53 overexpression in BMDMs. We found that USP53 overexpression reduced the ubiquitination level of PPARα. Moreover, analysis of ubiquitin linkage types indicated that K48-linked ubiquitination of PPARα was decreased, suggesting that USP53 may stabilize PPARα by antagonizing degradative ubiquitination(Fig. S8F). Taken together, these findings support a model in which FABP4 binds to PPARα and limits its interaction with USP53, thereby facilitating K48-linked polyubiquitination and proteasomal degradation of PPARα.

### Macrophage-specific FABP4 knockdown ameliorated HFD-induced MASH

The physiological role of macrophage FABP4 was further validated by adeno-associated virus (AAV)-mediated macrophage-specific FABP4 knock down in mice. To clarify the AAV experimental design, mice were randomly assigned to four groups: NCD+scramble, NCD+shFABP4, HFD+scramble, and HFD +shFABP4. We first verified its delivery efficiency and silencing efficacy in hepatic macrophages 30 days post-infection by immunofluorescence and by western blotting of gradient-centrifugation-isolated macrophages. Immunofluorescence analysis showed clear colocalization of GFP with F4/80-positive cells in liver sections, indicating successful delivery of the AAV construct to hepatic macrophages(Fig. S9A). In addition, western blot analysis of isolated liver macrophages confirmed that FABP4 protein expression was markedly reduced in the AAV-shFABP4 group compared with controls (Fig. S9B), whereas FABP4 expression in hepatocytes showed no significant difference between the two groups.(Fig. S9C). These data demonstrate that the AAV vector effectively targeted liver macrophages and achieved efficient FABP4 knockdown in vivo.

**Fig. 9:**
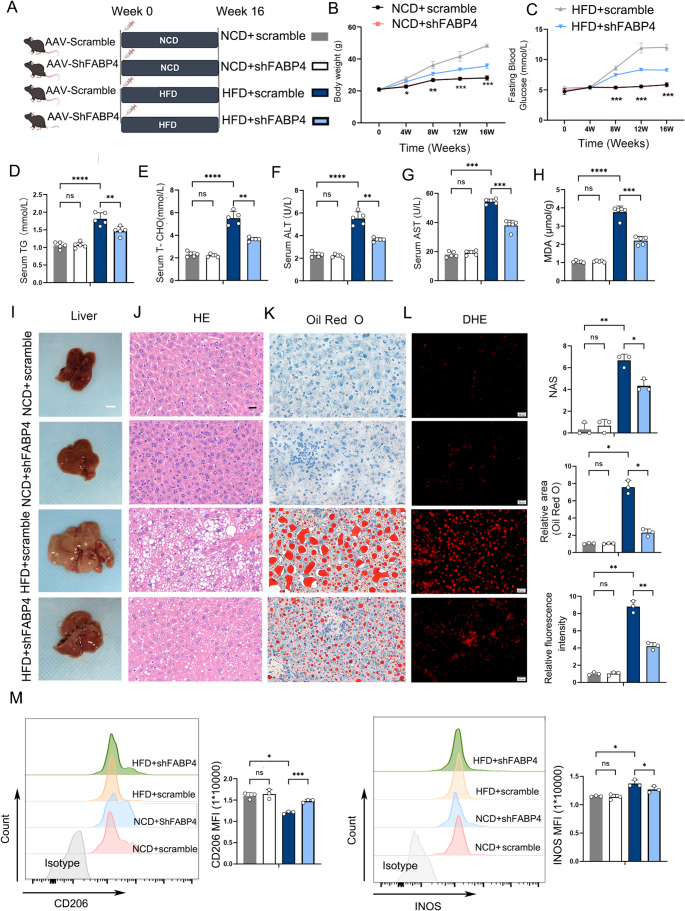
Macrophage-specific FABP4 knockdown ameliorated HFD-induced MASH. (**A**) Schematic diagram of the MASH model in mice following intravenous tail vein injection of adenovirus. (**B**) Body weight (*n* = 5) and (**C**) Fasting blood glucose levels (*n* = 5) in different groups. (**D**) Serum TG levels, (**E**) Serum total cholesterol (TC) levels(*n* = 5), (**F**) Serum alanine aminotransferase (ALT) levels(*n* = 5), (**G**) Serum aspartate aminotransferase (AST) levels(*n* = 5) and (**H**) Hepatic malondialdehyde (MDA) (*n* = 5). (**I**) Gross images of liver tissues. (**J**) H&E staining(*n* = 3). (**K**) ORO staining(*n* = 3) and (**L**) Dihydroethidium (DHE) staining (*n* = 3, scale bar, 20 μm). (**M**) Hepatic macrophages are isolated from the liver using gradient centrifugation. The proportions of iNOS+ macrophages and CD206 + macrophages are analyzed via flow cytometry with the respective antibodies. Data are presented as mean ± SD. In all scatter plots with bars, each data point represents one individual. *p < 0.05, **p < 0.01, ***p < 0.001, ****p < 0.0001

The mice were subsequently given a high-fat diet to establish the MASH model (Fig. 9A). Our results revealed that body weight and fasting blood glucose levels as well as glucose and insulin tolerance improved in the HFD +shFABP4 group mice than in the control group (Fig. 9B, C and Fig. S9D-G). Triglyceride (TG) and total cholesterol (TCHO) levels in serum were markedly lower in HFD +shFABP4 group mice than in controls (Fig. D, E). Additionally, FABP4 knockdown attenuated HFD-induced increases in ALT and AST levels (Fig. 9F, G). Furthermore, the level of malondialdehyde (MDA) in liver tissue also decreased significantly in HFD +shFABP4 group mice (Fig. 9H). Liver morphology and Histological analyses, including H&E staining, Oil Red O staining, and Dihydroethidium (DHE) staining, revealed reduced inflammation, lipid droplet and oxidative stress accumulation in the livers of macrophage-specific FABP4-knockdown mice subjected to HFD exposure (Fig. 9I-L). Compared with those from control mice, liver tissues from HFD +shFABP4 group mice presented significant decreased the proportion of iNOS+ macrophages while increasing the proportion of CD206 + macrophages in MASH mice (Fig. 9M). Together, these results demonstrate the close participation of FABP4 in MASH pathogenesis.

## Discussion

A previous study reported that patients with MASLD have higher plasma fibrin levels than healthy individuals [35]. Accumulating evidence indicates that proinflammatory macrophages drive MASLD progression, whereas anti-inflammatory macrophages exert protective effects [36, 37]. However, the relationship between fibrin and macrophages in MASH remains incompletely understood. In this study, we demonstrate that fibrin stiffness regulates macrophage polarization and lipid metabolic reprogramming, thereby influencing MASH progression. Notably, this mechano-immunometabolic mechanism differs from current MASH therapies, such as FXR and THR-β agonists, exemplified by obeticholic acid and resmetirom, which primarily act through hepatocyte-centered bile acid and metabolic signaling pathways [38, 39]. Our data suggest that fibrin stiffness acts upstream of macrophage polarization and reshapes the hepatic inflammatory microenvironment. Interestingly, the anti-inflammatory phenotype induced by soft fibrin may share functional similarities with beneficial macrophage subsets recently identified in human MASH, particularly TREM2⁺ lipid-associated macrophages that have been implicated in limiting disease progression [40]. These cells typically arise in lipid-rich and metabolically stressed microenvironments and are characterized by enhanced lipid handling, lysosomal activity, and reparative functions. In injured liver tissue, they are enriched in areas of inflammation and fibrosis and are thought to limit disease progression by suppressing excessive inflammatory activation and promoting tissue repair. However, we do not claim equivalence, as canonical markers of these subsets were not directly examined in the present study. Together, these findings suggest that modulation of fibrin mechanics may represent a complementary therapeutic strategy for MASH by promoting a protective macrophage state.

We observed increased fibrin deposition accompanied by enhanced infiltration of proinflammatory macrophages in the livers of patients with MASH and mice. As an extracellular matrix component, fibrin is an established spatial signal for innate immune cell clustering and the initiation of pathogenic inflammation in tissues [41, 42]. Here, we examined the effects of fibrin on macrophages. Using an in-house established 3D fibrin matrix, our data showed that soft fibrin, compared to stiff fibrin or standard culture flasks, suppressed proinflammatory cytokine expression while promoting anti-inflammatory marker expression. Consistently, Hsieh et al. reported that 2 mg/mL fibrin exerts a protective effect on macrophages [14]. IL-4 and IL-13 have been shown to promote fatty acid oxidation in M2 macrophages [43, 44]. We found that lipid accumulation and TG content in macrophages were substantially reduced by soft fibrin upon extracellular lipid loading. Although fibrin deposition was increased in both human and murine MASH livers, we did not directly measure fibrin structure or local mechanical properties in vivo. Thus, our study does not demonstrate that fibrin stiffness is altered during MASH progression or that fibrin mechanics are the dominant determinant of liver stiffness in the context of collagen-rich fibrosis. Our 3D fibrin model was used as a mechanistic platform to examine whether fibrin stiffness can regulate macrophage polarization and lipid metabolic reprogramming in a fibrin-enriched microenvironment. Future studies combining spatial analysis of fibrin architecture with direct biomechanical measurements will be needed to define the in vivo contribution of fibrin mechanics to MASH progression.

FABP4 plays a crucial role in metabolism and inflammation, including in rheumatoid arthritis [45], diabetes [30], and pancreatic cancer [46]. FABP4 levels have been identified as a predictive factor for poor prognosis in patients with MASLD [47], however, the underlying mechanism remains unclear. Our data demonstrated that macrophages cultured in soft fibrin exhibited lower FABP4 protein expression than those cultured in stiff fibrin or flasks. Furthermore, soft fibrin modulated the anti-inflammatory polarization of macrophages through FABP4. Although the interaction between ITGB2 and FABP4 remains elusive. In this study, we demonstrated that ITGB2 regulates STAT1 phosphorylation in macrophages, and STAT1 serves as a critical transcription factor for FABP4. However, it is important to note that ITGB2 (CD18) is typically a part of the αMβ2 integrin (Mac-1) complex. Our study did not explore whether fibrin directly binds to Mac-1 in this system. Future research focusing on this interaction will provide additional insights into the role of Mac-1 in macrophage polarization and the potential therapeutic implications in metabolic dysfunction-related diseases. And although our data support an important role for the ITGB2/STAT1 axis in mediating the effects of fibrin stiffness on macrophage polarization and metabolic reprogramming, matrix stiffness sensing is likely to be more complex and may also involve additional mechanotransduction pathways, such as focal adhesion signaling, cytoskeletal tension, and YAP/TAZ-related signaling. Future studies will be needed to further dissect the contribution and potential crosstalk of these canonical mechanotransduction pathways in macrophage responses to fibrin stiffness.

PPARs are transcription factors that function as fatty acid receptors and regulate gene expression in response to fatty acid-derived stimuli [48]. PPARα is the most abundant isotype in hepatocytes and plays a crucial role in various aspects of lipid metabolism. Deletion of hepatocyte-restricted PPARα is sufficient to promote steatosis [49–51]. The role of the PPAR signaling pathway in M2 polarization likely involves a predominant enhancement of fatty acid β-oxidation and mitochondrial biogenesis at the transcriptional level [52]. In this study, we elucidate the molecular mechanism underlying PPARα polyubiquitination-mediated degradation. Our findings provide a new perspective, demonstrating that PPARα turnover is regulated by the ubiquitin-proteasome system. Mechanistically, we show that FABP4 blocks USP53-mediated deubiquitination, thereby increasing PPARα ubiquitination and degradation. Thus, this study expands the spectrum of biological activities regulated by FABP4 and highlights its role in lipid metabolism through PPARα regulation.

Furthermore, tail vein injection of macrophages cultured in soft fibrin led to substantial remission of hepatic steatosis and liver injury, along with reduced fibrin and FABP4 deposition in MASH mice. In contrast, macrophages cultured in stiff fibrin had no therapeutic effect. These results suggest that fibrin stiffness contributes to hepatic steatosis and liver injury, at least in part, by modulating macrophage lipid metabolism. More importantly, FABP4 expression was significantly inhibited following the infusion of soft fibrin-cultured macrophages into MASH mice. In agreement with our findings, Jeong et al. reported that Korean red ginseng alleviates MASH-related inflammatory responses by inhibiting FABP4 expression in vivo and in vitro [53]. To further investigate the role of FABP4 in MASH, we generated macrophage-targeted FABP4 knockdown mice through intrahepatic delivery of adeno-associated virus 8 (AAV8) carrying shFabp4. The mouse model demonstrated that macrophage-specific FABP4 knockdown ameliorated HFD-induced MASH.

In conclusion, our study demonstrates that soft fibrin promotes the anti-inflammatory polarization of macrophages and enhances fatty acid β-oxidation through FABP4 downregulation, thereby alleviating hepatic steatosis, insulin resistance, inflammation and oxidative stress in MASH mice. These findings suggest that pharmacological modulation of fibrin remodeling or stiffness, as well as repurposing FABP4-targeted inhibitors, may represent potential strategies to mimic the beneficial effects of soft fibrin. Our study identifies a macrophage-centered Fibrin-FABP4-PPARα axis that may complement existing hepatocyte-centered therapies for MASH.

### Limitations and future directions

However, several limitations of this study should be acknowledged. First, although the HFD-fed mouse model recapitulated key metabolic and histological features of MASH, it does not fully reflect the heterogeneity of human disease. In addition, the use of only male mice, together with the relatively small sample size and limited demographic diversity of the human cohort may restrict the generalizability of our conclusions. Second, the translational feasibility of modulating fibrin stiffness in vivo remains uncertain, as fibrin stiffness in this study was manipulated in vitro by altering fibrinogen concentrations, which may not fully recapitulate the complex mechanical microenvironment of human MASH liver tissue, the spatiotemporal dynamics of fibrin deposition and remodeling during human MASH progression remain unclear. Moreover, although soft fibrin-cultured macrophages exerted therapeutic effects in vivo, adoptive macrophage transfer still faces practical challenges, including variable homing efficiency, potential phenotypic instability after transfer, and limited clinical scalability. Third, although the transferred macrophages predominantly accumulated in the liver, a small proportion was also detected in other organs, suggesting that potential systemic effects cannot be completely excluded. Finally, our analysis focused on the overall hepatic macrophage population and did not distinguish resident Kupffer cells from monocyte-derived macrophages, which may have distinct roles during MASH progression. Future studies using human primary macrophages or liver organoid systems will be needed to further validate the translational relevance of our findings.

## Supplementary Information

Below is the link to the electronic supplementary material.

Supplementary file1 (DOCX 25.3 kb)

Supplementary file2 (XLSX 441 kb)

Supplementary figure 1 (PNG 989 kb)

High Resolution Image (TIF 1.22 mb)

Supplementary figure 2 (PNG 247 kb)

High Resolution Image (TIF 383 kb)

Supplementary figure 3 (PNG 2.55 mb)

High Resolution Image (TIF 3.40 mb)

Supplementary figure 4 (PNG 2.21 mb)

High Resolution Image (TIF 2.98 mb)

Supplementary figure 5 (PNG 1.42 mb)

High Resolution Image (TIF 1.83 mb)

Supplementary figure 6 (PNG 314 kb)

High Resolution Image (TIF 444 kb)

Supplementary figure 7 (PNG 1.25 mb)

High Resolution Image (TIF 1.64 mb)

Supplementary figure 8 (PNG 1.03 mb)

High Resolution Image (TIF 1.33 mb)

Supplementary figure 9 (PNG 1.25 mb)

High Resolution Image (TIF 1.54 mb)

## Data Availability

The IP-MS dataset has been deposited to the ProteomeXchange Consortium via the PRIDE partner repository under the dataset identifier PXD077311. The metabolomics data supporting the findings are provided in Supplementary Table S6. Additional metabolomics data are available from the corresponding author upon reasonable request.
