# Supplementary material for: Soft fibrin matrix instructs macrophage M2 polarization via FABP4-mediated enhancement of fatty acid β-oxidation to alleviate MASH
Source: Cell Mol Life Sci. 2026 Jun 4;83(1):299. doi: 10.1007/s00018-026-06266-2 (PMC13452796; doi:10.1007/s00018-026-06266-2)
Supplement: Supplementary file 1 [file 18_2026_6266_MOESM1_ESM.docx]

**Supplementary Table 1.** Baseline clinical characteristics of the study subjects.

| **Patients No.** | **Age (years)** | **Gender** | **diabetes** | **BMI (kg/m^2^)** | **NAS（score）** | **Fibrosis (stage)** |
| --- | --- | --- | --- | --- | --- | --- |
| 1 | 59 | Female | No | 31.8 | 4 | 0 |
| 2 | 57 | Female | No | 28.3 | 6 | 1 |
| 3 | 64 | Male | No | 29.1 | 5 | 1 |
| 4 | 53 | Male | Yes | 27.8 | 3 | 0 |
| 5 | 60 | Female | No | 28.0 | 5 | 0 |
| 6 | 55 | Male | Yes | 31.6 | 4 | 0 |
| 7 | 46 | Female | No | 31.2 | 3 | 0 |
| 8 | 57 | Male | No | 30.3 | 6 | 1 |
| 9 | 49 | Female | Yes | 29.1 | 3 | 0 |
| 10 | 61 | Male | No | 32.8 | 6 | 0 |
| 11 | 47 | Male | No | 29.2 | 4 | 0 |
| 12 | 55 | Female | Yes | 30.6 | 6 | 1 |

**Supplementary Table 2**.siRNA sequences

| **SiRNA** | **Species** | **Sence (5'-3')** |
| --- | --- | --- |
| SiFABP4#1 | mouse | GGGCTTTGCCACAAGGAAA |
| SiFABP4#2 | mouse | CCAAGCCCAACATGATCAT |
| SiFABP4#3 | mouse | GGTGGAATGTGTTATGAAA |
| SiITGB2#1 | mouse | CCAGGAAUGCACCAAGUACAA |
| SiITGB2#1 | mouse | CGGAAGGAACAUUUACAACAU |
| SiITGB2#1 | mouse | GCAAUAAUGGAGCAUCGAGUA |

**Supplementary Table 3**. Primers for qPCR

| **Gene** | **Species** | **Sequence 5’-3’** |
| --- | --- | --- |
| Fabp4-Forward | mouse | TGAAATCACCGCAGACGACAGG |
| Fabp4-Reverse | mouse | GCTTGTCACCATCTCGTTTTCTC |
| Pparα-Forward | mouse | ACCACTACGGAGTTCACGCATG |
| Pparα-Reverse | mouse | GAATCTTGCAGCTCCGATCACAC |
| Cpt1a-Forward | mouse | GGCATAAACGCAGAGCATTCCTG |
| Cpt1a-Reverse | mouse | CAGTGTCCATCCTCTGAGTAGC |
| Fasn-Forward | mouse | CACAGTGCTCAAAGGACATGCC |
| Fasn-Reverse | mouse | CACCAGGTGTAGTGCCTTCCTC |
| Srebf1-Forward | mouse | CGACTACATCCGCTTCTTGCAG |
| Srebf1-Reverse | mouse | CCTCCATAGACACATCTGTGCC |
| Cd36-Forward | mouse | GGACATTGAGATTCTTTTCCTCTG |
| Cd36-Reverse | mouse | GCAAAGGCATTGGCTGGAAGAAC |
| Acly-Forward | mouse | AGGAAGTGCCACCTCCAACAGT |
| Acly- Reverse | mouse | CGCTCATCACAGATGCTGGTCA |
| iNos- Forward | mouse | GAGACAGGGAAGTCTGAAGCAC |
| iNos- Reverse | mouse | CCAGCAGTAGTTGCTCCTCTTC |
| Arg1-Forward | mouse | CATTGGCTTGCGAGACGTAGAC |
| Arg1-Reverse | mouse | GCTGAAGGTCTCTTCCATCACC |
| Tnfα-Forward | mouse | GGTGCCTATGTCTCAGCCTCTT |
| Tnfα-Reverse | mouse | GCCATAGAACTGATGAGAGGGAG |
| Cd206-Forward | mouse | GTTCACCTGGAGTGATGGTTCTC |
| Cd206-Reverse | mouse | AGGACATGCCAGGGTCACCTTT |
| Itgb2-Forward | mouse | CTTTCCGAGAGCAACATCCAGC |
| Itgb2-Reverse | mouse | GTTGCTGGAGTCGTCAGACAGT |
| gapdh-Forward | mouse | CATCACTGCCACCCAGAAGACTG |
| gapdh-Reverse | mouse | ATGCCAGTGAGCTTCCCGTTCAG |

**Supplementary Table 4**.The antibody used in Western blot.

| **Reagent** | **Company** | **Catalog #** |
| --- | --- | --- |
| Anti-FABP4 | Proteintech | 12802-1-AP |
| Anti-PPARα | abcam | ab314112 |
| Anti-CPT1A | Proteintech | 15184-1-AP |
| Anti-iNOS | Proteintech | 18985-1-AP |
| Anti-TNFα | Zenbio | 346654 |
| Anti-CD206 | Proteintech | 18704-1-AP |
| Anti-Arg1 | Proteintech | 16001-1-AP |
| Anti-SREBP1 | Proteintech | 14088-1-AP |
| Anti-FASN | Cell Signaling Technology | 3180 |
| Anti-CD36 | Proteintech | 18836-1-AP |
| Anti-GAPDH | Proteintech | 60004-1-Ig |
| Anti-ACLY | Proteintech | 15421-1-AP |
| Anti-DYKDDDDK Tag | Proteintech | 66008-4-Ig |
| Anti-His-tag mAb | ABclonal | AE086 |
| Anti-Fibrin | Thermo Scientific | ZA4131161 |
| HRP-conjugated Affinipure Goat Anti-Rabbit IgG(H+L) | Proteintech | SA00001-2 |
| Anti-HUWE1 | HUABIO | HA721815 |
| Anti-USP53 | Immunoway | YT4845 |
| Anti-Rabbit IgG | Cell Signaling Technology | 2729S |
| Anti-ITGB1 | Zenbio | 310144 |
| Anti-ITGB2 | ABclonal | A25810 |
| Anti-ITGB3 | Proteintech | 18309-1-AP |
| Anti-ITGB4 | ABclonal | [A4596](https://abclonal.com.cn/catalog/A4596) |
| Anti-ITGB5 | santa cruz | sc-398214 |
| Anti-ITGB6 | ABclonal | [A16904](https://abclonal.com.cn/catalog/A16904) |
| Anti-ITGB7 | Zenbio | 252524 |
| Anti-ITGB8 | Affnity Biosciences | #DF2545 |
| Anti-STAT1 | Cell Signaling Technology | 14994s |
| Anti-DYKDDDDK Magnetic Beads | Biolinkedin | L1011 |
| Anti-His Magnetic Beads | Biolinkedin | L1015 |
| Goat anti-mouse/rabbit poly-HRP secondary antibody | Proteintech | PR30009 |

**Supplementary Table 5**. The antibody used in flow cytometry.

| **Reagent** | **Company** | **Catalog #** |
| --- | --- | --- |
| F4/80 | BioLegend | 123116 |
| CD206 | BioLegend | 141703 |
| CD16/32 | BioLegend | 101319 |
| iNOS | BioLegend | 696805 |
| CD45 | BioLegend | 103130 |
| CD11b | BioLegend | 101206 |
